# Inversion of the Complex Refractive Index of Au-Ag Alloy Nanospheres Based on the Contour Intersection Method

**DOI:** 10.3390/ma16093291

**Published:** 2023-04-22

**Authors:** Long Cheng, Paerhatijiang Tuersun, Dengpan Ma, Dilishati Wumaier, Yixuan Li

**Affiliations:** Xinjiang Key Laboratory for Luminescence Minerals and Optical Functional Materials, School of Physics and Electronic Engineering, Xinjiang Normal University, Urumqi 830054, China

**Keywords:** Mie theory, contour intersection method, Au-Ag alloy nanospheres, complex refractive index

## Abstract

The contour intersection method is a new method used to invert the complex refractive index of small particles. Research has yet to be reported on using this method to invert the complex refractive index of nanoparticles. This paper reports the feasibility and reliability of the contour intersection method in the inversion of the complex refractive index of nanoparticles using Au-Ag alloy nanospheres. The Mie theory and the size-dependent dielectric function are used to calculate the light scattering and absorption efficiency of Au-Ag alloy nanospheres corresponding to the complex refractive index. The complex refractive index of the particles is obtained by inversion with the contour intersection method. The backscattering efficiency constraint method is used to determine the unique solution when multiple valid solutions from the contour intersection method appear. The effects of the Au component percentage, particle size, and measurement errors on the inversion results are quantitatively analyzed. Finally, the inversion accuracy is compared and analyzed with the traditional iterative method. The results show that as long as the light scattering efficiency, light absorption efficiency, and backscattering efficiency of Au nanospheres can be measured, the accurate complex refractive index can also be calculated by inversion using the contour intersection method. The accuracy of the inversion results can be ensured when the measurement error is less than 5%. The results of inversion using the contour intersection method are better than those of the iterative methods under the same conditions. This study provides a simple and reliable inversion method for measuring the complex refractive index of Au-Ag alloy nanospheres.

## 1. Introduction

Noble metal nanoparticles are the most widely studied colloidal system in nanoscience and nanotechnology [1]. Localized surface plasmon resonance (LSPR) can occur when metal nanoparticles interact with incident light at specific wavelengths, called resonant wavelengths [2]. At the resonant wavelengths, nanoparticles strongly absorb and scatter incident light [3,4]. The resonant wavelength of the metal nanoparticles can be tuned from near-ultraviolet to near-infrared [5,6], making metal nanoparticles suitable in many fields such as bioimaging [7], photothermal therapy [8], surface-enhanced Raman scattering (SERS) [9], biosensing, and detection [10,11].

With the rapid development of nanotechnology, it is now possible to design and study different composite nanoparticles for various applications. Composite nanoparticles are widely studied because they overcome the deficiency of ordinary nanoparticles. Moreover, composite nanoparticles possess new properties arising from the synergistic effects of the combination in addition to the properties of their constituents [12,13,14]. Au nanoparticles have good chemical stability and photothermal properties and exhibit stable plasmonic reactions. Ag nanoparticles, on the other hand, have high refractive index sensitivity, strong surface-enhanced Raman scattering, and photocatalytic activity [15,16]. With the advantages of Au and Ag, Au-Ag alloy nanoparticles have been widely studied due to their excellent structural stability, biocompatibility, photothermal properties, refractive index sensing, strong surface-enhanced Raman scattering, and potential biomedical applications [17,18]. It is found that the solar thermal conversion efficiency of Au-Ag alloy nanoparticles is higher than that of gold nanoparticles [15]. Hollow Au-Ag alloy nanoparticles show better oxidation resistance [19]. Au-Ag alloy nanoparticles have higher refractive index sensitivity and surface-enhanced Raman scattering than gold nanoparticles [20]. Au-Ag alloy nanoparticles of different sizes and shapes can be prepared by synthesis methods such as chemical reduction techniques, nanosecond laser-induced alloying, and femtosecond laser irradiation [21]. Alloy nanoparticles provide a solution to the problems of monometallic nanoparticles in practical applications.

The complex refractive index is one of the important optical properties of nanoparticles used in many practical applications [22,23]. Therefore, accurately measuring the complex refractive index of Au-Ag alloy nanoparticles has essential research significance and value. Currently, the commonly used measurement methods include ellipsometry, laser transmission, dynamic and static light scattering, and light extinction spectroscopy [24,25,26,27]. Although ellipsometry measurements are accurate and can be monitored in real-time, it is challenging to characterize low absorption coefficients with this technique [24]. The measurement accuracy in the laser transmission method is directly related to the accuracy of the experimental instrument [25]. The dynamic and static light scattering method has no requirements for the dispersion of the sample and is suitable for particles from micron to submicron. However, the relevant experimental setup is complex [26]. The light extinction spectrometer measures the extinction coefficient in extinction spectroscopy. Then, Mie theory is used to invert the complex refractive index of particles. However, the light extinction spectroscopy method also has the limitation that the inversion error will increase when the concentration of the particle system is too high [27]. Mie theory is used to calculate the optical properties of nanoparticles such as Au-Ag alloys, Co-Plus-NI doped ZnO, and Ni/Cu co-doped ZnO [28,29]. The contour intersection method is similar to the light extinction spectroscopy, in which the scattering efficiency, absorption efficiency, and backscattering efficiency of a particle are measured first. Then, the complex refractive index of the particle is inverted using Mie theory [30]. The inversion method is a technique for determining the complex refractive index of a nanomaterial by measuring the scattering and absorption efficiencies of light at different wavelengths. In the inversion method, the measured scattering and absorption data are used to determine the complex refractive index of the material. This quantity is often denoted as *n* + i*k*, where *n* and *k* are the real and imaginary parts of the refractive index, respectively. The inverted complex refractive index is a valuable parameter for characterizing the optical properties of materials, including their absorption, scattering, and dispersion behavior. It can also be used to derive other optical constants such as the extinction coefficient and dielectric function. Compared to simulation and fitting procedures, the inversion method has several advantages. It is a physically based approach that does not require assumptions about the underlying model or functional form of the data. It is also relatively fast and accurate for certain types of materials and structures, especially when the material parameters are well-defined and homogeneous. However, the inversion method may not be suitable for complex or heterogeneous samples. In such cases, simulation and fitting procedures that incorporate more detailed models and empirical fitting parameters may be preferred.

Among the many complex refractive index measurement methods, the contour intersection method is of great interest because of its simplicity, low cost, real-time visual observation of the inversion process, and ability to quickly obtain the complex refractive index of particles [30]. Dapeng Zhao et al. [31] calculated the aerosol equivalent complex refractive index using the observed aerosol scattering coefficients, absorption coefficients, and particle size distribution data, providing more data support for future modeling in the Taishan area. Aidan Rafferty et al. [32] calculated the size and complex refractive index of aqueous aerosol particles using electromagnetic heating and cavity-enhanced Raman scattering. Renju Nandan et al. [33] estimated the complex refractive index of aerosol over a tropical atmosphere using a synergy of in situ measurements. As can be seen, the studies mentioned above have measured complex refractive indices of atmospheric aerosols. However, measurements of the refractive indices of other particles, especially gold and silver alloy nanoparticles, have rarely been reported.

In this paper, combined with Mie theory, an improved contour intersection method is proposed to measure the complex refractive index of Au-Ag alloy nanoparticles. The feasibility of the inversion results and the factors affecting the inversion results are quantitatively analyzed. The proposed method provides a fast and effective way to measure the complex refractive index of Au-Ag alloy nanoparticles.

## 2. Contour Intersection Method

**Figure 1:**
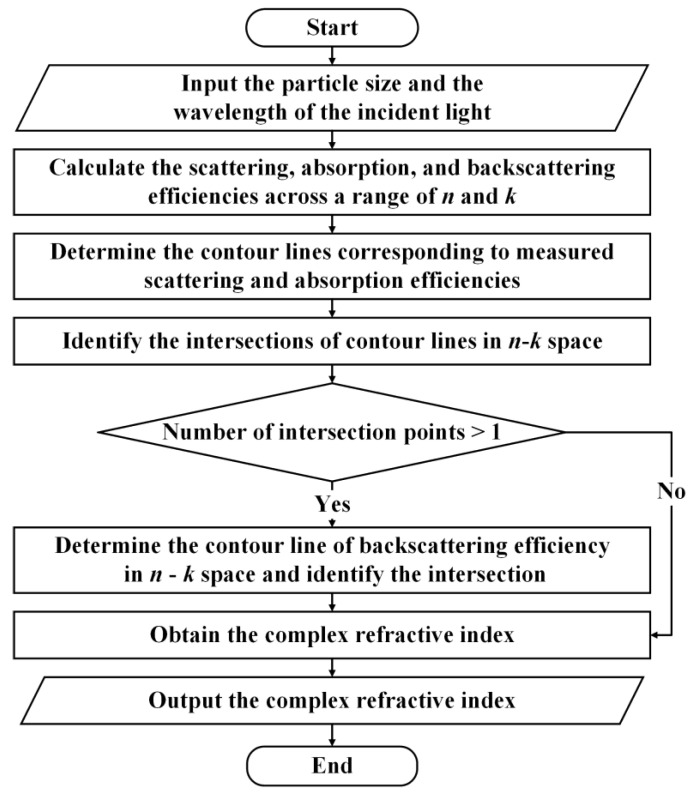
Flowchart of the inversion process of the contour intersection method.

The contour intersection method visualizes various optical parameter spaces as functions of *n* and *k* and looks for intersections in the curves defined by optical measurements. [30] The specific process of this method is shown in Figure 1. Firstly, the light scattering, absorption and backscattering efficiencies of nanoparticles with a given wavelength and diameter across a range of *n* and *k* are calculated. Then, the contours corresponding to measured scattering and absorption efficiencies are determined. Finally, intersection points on the contour line of the scattering and absorption efficiencies in n-k space are identified. If there is only one intersection point, the result is output, and the relative error is calculated. If multiple intersection points exist, the backscattering efficiency is added to constrain the unique solution. The scattering, absorption, and backscattering efficiencies of particles can be calculated using Mie theory.

**Figure 2:**
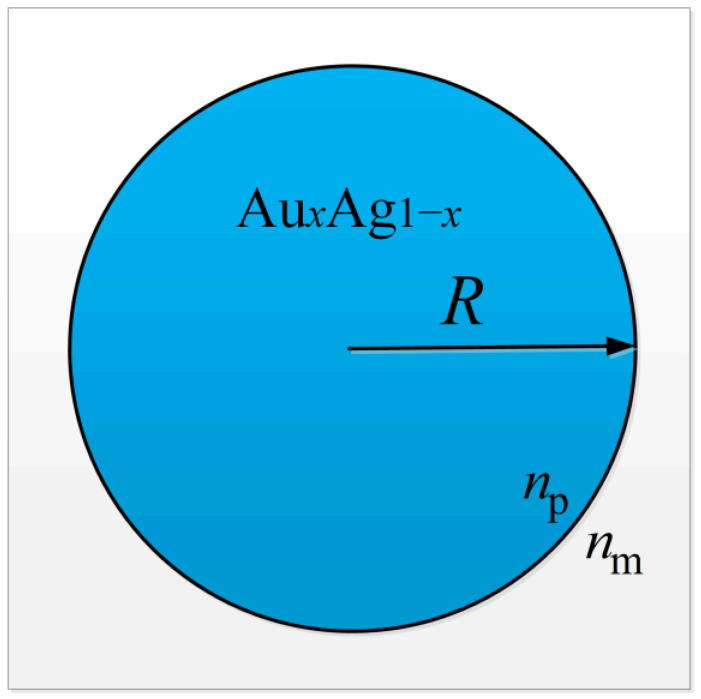
Geometry of Au-Ag alloy nanospheres.

For the Au-Ag alloy nanospheres, *R* represents the particle radius, *x* represents the molar fraction of gold, *n*_p_ is the particle refractive index, and *n*_m_ is the refractive index of the surrounding medium, as shown in Figure 2.

When a beam of light irradiates the particles, LSPR occurs on the surface, and particles strongly scatter and absorb light. The scattering and absorption efficiency of particles can be calculated using Mie theory [34]. Detailed descriptions and derivations have been published previously [35,36,37]. The expressions for extinction, scattering, absorption, and backscattering efficiencies of spherical nanoparticles are as follows.

(1) Qext=2x2∑n=1∞(2n+1)Re(an+bn) 

(2) $$Q_{sca}=\frac{2}{x^{2}}\sum_{n=1}^{\infty }\left(2n+1)(|a_{n}{|}^{2}+|b_{n}{|}^{2}\right)$$

(3) Qabs=Qext−Qsca=2x2∑n=1∞(2n+1)[Re(an+bn)−|an|2−|bn|2]

(4) $$Q_{back}=\frac{1}{x^{2}}{\left|\sum_{n=1}^{\infty }\left(2n+1){\left(-1\right)}^{n}(a_{n}-b_{n}\right)\right|}^{2}$$

Here, *x* = 2*πRn_m_/λ* is the size parameter, and *λ* is the wavelength of incident light in a vacuum. The numerical algorithm described in Bohren and Huffman’s monograph [35] was used to calculate scattering coefficients *a_n_* and *b_n_*. As seen from the Equations (1)–(4), to solve the interaction problem between light and spherical particles, Mie theory only needs to know the incident light wavelength, particle diameter, particle refractive index, and refractive index of the surrounding medium. The calculations of extinction, scattering, and absorption efficiencies of spherical particles are then straightforward.

The refractive index of the particles can be calculated by the dielectric function while calculating the extinction, scattering, absorption, and backscattering efficiencies. Since the Au-Ag alloy nanoparticles are nonmagnetic, *μ_r_* = 1, the refractive index of the nanoparticles can be written as follows.

(5) $$n=\sqrt{\varepsilon_{r}}$$

where *ε_r_* is the dielectric function.

In this study, an alloy dielectric function model developed by Rioux et al. [36] is adopted, which includes the Drude term and two critical points. This model is related to both optical frequency and alloy composition. It also considers the effect of particle size and alloy ratio on the dielectric function. The dielectric function of alloy nanoparticles can be expressed as follows.

(6) $$\varepsilon r\left(\omega ,x\right)=\varepsilon_{\infty }-\frac{{\left(\omega_{p}\left(x\right)\right)}^{2}}{\omega^{2}+i\omega \Gamma_{p}\left(x\right)}+\varepsilon_{\mathrm{CP}1}\left(\omega ,\omega_{01}\left(x\right),\omega_{g1}\left(x\right),\Gamma_{1}\left(x\right),A_{1}\left(x\right)\right)+\varepsilon_{\mathrm{CP}2}\left(\omega ,\omega_{02}\left(x\right),\Gamma_{2}\left(x\right),A_{2}\left(x\right)\right)$$

where *ω* is the angular frequency of the incident light, and *x* is the mole fraction of Au. The mole fraction of Ag is 1 − *x*. *ε_∞_* is the contribution of free electrons from high energy level transition. *ω*_p_ is the plasma frequency, and Γ_p_ is the damping coefficient of free electrons (collision frequency). *ε*_CP1_ and *ε*_CP2_ are the dielectric functions at two critical points. *ω*_01_ and *ω*_02_ are the transition thresholds at two critical points. *ω*_g1_ is the transition gap at the critical point. Γ_1_ and Γ_2_ are the damping coefficients at two critical points. *A*_1_ and *A*_2_ are amplitude parameters at two critical points.

Each parameter in the equation is related to the alloy composition. The frequency of the alloy plasma with any Au mole fraction can be written as follows.

(7) $$\omega_{p}\left(x\right)=x^{2}\left(2\omega_{\mathrm{pAu}}-4\omega_{\mathrm{pAuAg}5050}+2\omega_{\mathrm{pAg}}\right)+x\left(-\omega_{\mathrm{pAu}}+4\omega_{\mathrm{pAuAg}5050}-3\omega_{\mathrm{pAg}}\right)+\omega_{\mathrm{pAg}}$$

where *ω*_pAu_, *ω*_pAg_, and *ω*_pAuAg5050_ represent the plasma frequencies of pure Au, pure Ag, and 50%–50% equimolar fraction Au-Ag alloys, respectively. The other parameters also have the same composition dependence. In the above dielectric function model (Equation (6)), Rioux et al. used a genetic algorithm to fit multiple sets of experimental measurement data to obtain all unknown parameters in the model. The fitting parameters are shown in Table 1.

The collision frequency Γ_p_ of free electrons in metal nanoparticles is related to the average free path of free electrons. Similarly, Γ_p_ in Au-Ag alloy nanoparticles is also affected by the mean free path of free electrons. Therefore, the dielectric function of alloy nanoparticles can be modified as follows [37].

(8) $$\Gamma_{p}=\Gamma_{\mathrm{pBulk}}+\alpha \frac{hv_{f}}{L_{\mathrm{eff}}}$$

where Γ_pBulk_ is the damping coefficient of bulk material, which can be calculated by fitting parameters. *α* is a dimensionless parameter close to 1. *h* is the Planck constant. *v_f_* is the Fermi rate of free electrons, which is 1 × 10^6^ m/s for Au-Ag alloys [36]. *L*_eff_ represents the effective mean free path of free electrons, the size of nanoparticles [38].

## 3. Results and Discussion

**Figure 3:**
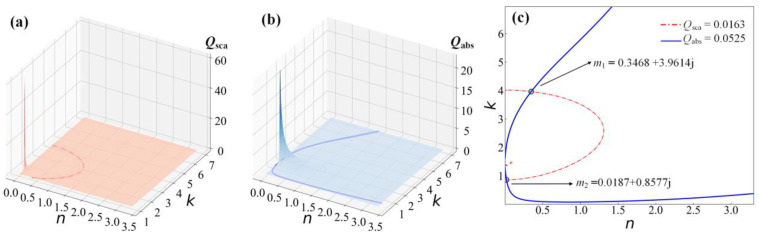
The complex refractive index of Au nanoparticles with diameters of 50 nm at a wavelength of 633 nm. (**a**) Scattering efficiency (red). (**b**) Absorption efficiency (blue). (**c**) Scattering and absorption efficiencies after isometric projection on the n-k plane are inverted by the contour intersection method.

We performed numerical simulations using the contour intersection method to obtain the composite refractive index of Au-Ag alloy nanoparticles. The process of contour line intersection inversion is shown in Figure 3. In the absence of *E*_m_, the measurement error, it is assumed that the diameters of the test particles are 50 nm, the incident wavelength is 633 nm, and *x* is 0.62. The original value of the particle complex refractive index (*n*_o_, *k*_o_) is calculated by the modified model in the range *n* ∈ [*n*_o_ − ∆*n*, *n*_o_ + ∆*n*]; *k* ∈ [*k*_o_ − ∆*k*, *k*_o_ + ∆*k*]; ∆*n* = ∆*k* = 3 and with a step length of 0.001. The scattering efficiency (*Q*_sca_) and absorption efficiency (*Q*_abs_) in a given complex refractive index range are calculated by Mie theory, as shown in Figure 3a,b. Then, the contour lines are drawn using *Q*_sca_ and *Q*_abs_ corresponding to the original values of the complex refractive index. The two contour lines are projected onto the *n-k* plane to find their intersection, as shown in Figure 3c.

**Figure 4:**
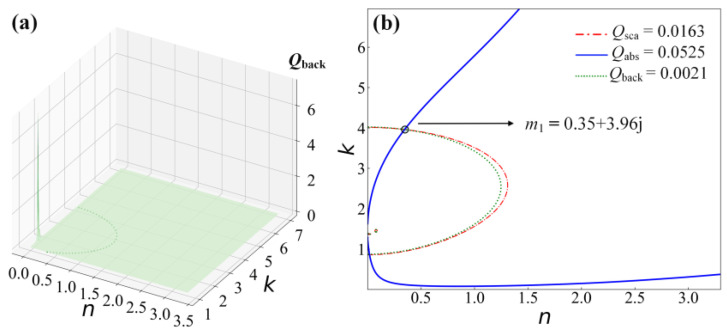
The complex refractive index of Au nanoparticles with diameters of 50 nm at 633 nm. (**a**) Backscattering efficiency (green). (**b**) Projection of contour lines in the *n-k* plane inverted by the contour intersection method.

In order to solve the problem of multiple inversion effective solutions when only *Q*_sca_ and *Q*_abs_ are specified in the actual operation, the contour line of *Q*_back_ is added to *n*-*k* space, as shown in Figure 4a. Finally, the contours of *Q*_sca_, *Q*_abs_, and *Q*_back_ are identified and projected onto the *n-k* space. The results show that adding *Q*_back_ can acquire a unique solution, as shown in Figure 4b.

The Au-Ag alloy nanospheres with 50 nm diameters were irradiated by 633 nm visible light. The complex refractive index was inverted by the contour line intersection method and traditional iterative method, as shown in Table 2. The original values of real and imaginary parts of the complex refractive index, the values of inversed complex refractive index, and their relative errors are given in the table. It can be seen from Table 2 that under this condition, the contour intersection method has ideal inversion results. However, how the changes in the proportion and sizes of Au components affect the accurate characterization of the inversion results requires further investigation.

Let us quantitatively analyze the influence of the Au component ratio, particle size, and measurement error on the complex refractive index inversion results of Au-Ag alloy nanospheres. The inversion accuracy is compared with that of the traditional iterative method. The accuracy and reliability of the inversion method are analyzed with the relative error of the real and imaginary parts of the complex refractive index. The relative error calculation formula is as follows.

(9) $$E_{n}=100\%\times \left|\frac{n_{i}-n_{o}}{n_{o}}\right|,\;E_{k}=100\%\times \left|\frac{k_{i}-k_{o}}{k_{o}}\right|$$

*E_n_* and *E_k_* are the relative errors in the real and imaginary parts of the complex refractive index. *n*_i_ and *n*_0_ are the inversion value and original value of the real part of the complex refractive index. *k*_i_ and *k*_o_ represent the inverse value and the original value of the imaginary part of the complex refractive index.

**Figure 5:**
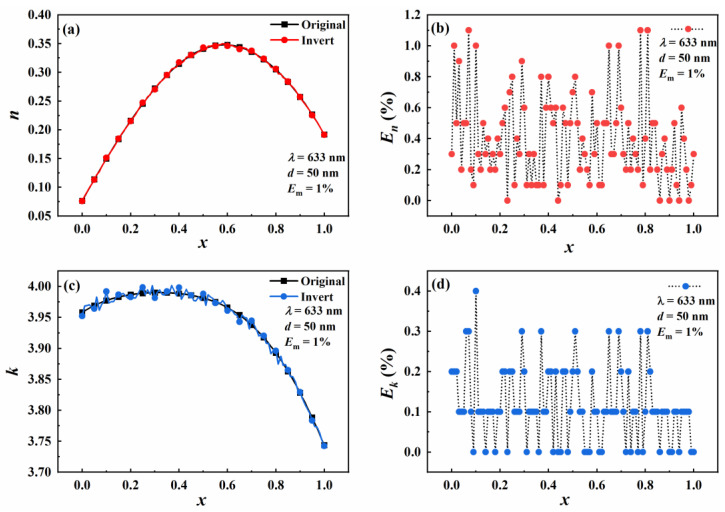
Effect of Au component proportion on the real part (*n*) and imaginary part (*k*) of the inverted refractive index (**a,c**) with the corresponding relative errors (**b,d**).

The following values of the parameters are used in the contour intersection method. The wavelength is 633 nm, the step size is 0.001, ∆*n* and ∆*k* are 0.2, and the particle diameters are 50 nm. Since the measurement errors in the actual measurement of *Q*_sca_ and *Q*_abs_ cannot be avoided, [−1%, 1%] random errors are added to *Q*_sca_ and *Q*_abs_ at different wavelengths. *E*_m_ expresses random errors. The inversion results are shown in Figure 5.

Figure 5a,c shows the inversion results of the real and imaginary parts of the complex refractive index. It can be seen that the real part of the complex refractive index increases first and then decreases with the increase of the proportion of the Au molar fraction. The value of the real part of the complex refractive index reaches the maximum at 0.58. The imaginary part of the complex refractive index increases slowly and then decreases significantly with the increase of the proportion of the Au molar fraction. The value of the imaginary part of the complex refractive index reaches the maximum at 0.29. Figure 5b presents the relative error in the inversion of the real part of the complex refractive index. It can be seen that the relative error of the real part n of the complex refractive index is less than 1.2%. Figure 5d shows the relative error in the inversion of the imaginary part of the complex refractive index. It can be seen that the relative error in the imaginary part of the complex refractive index fluctuates steadily with the increase of the proportion of Au molar fraction and is less than 0.4%.

**Figure 6:**
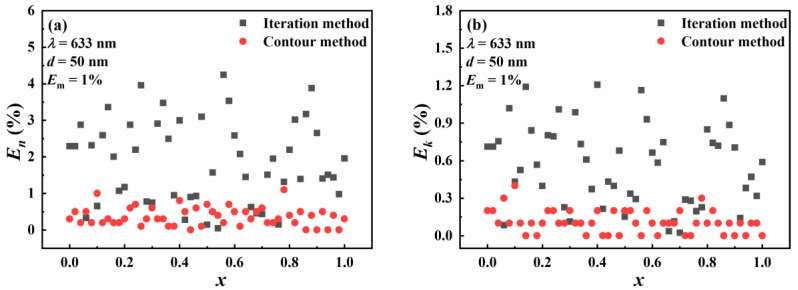
The relative errors in the real part (**a**) and imaginary part (**b**) of the complex refractive index inverted by the contour intersection method and the iterative method with respect to the change in the Au molar fraction.

The inversion results of the contour intersection method are compared with the traditional iterative method to see the influence of the proportion of Au on the inversion results and inversion accuracy more intuitively. The relative errors of the inversion results of the two methods are shown in Figure 6. It can be seen from Figure 6a,b that the relative error of the iterative method is as high as 4.3%. However, the relative error of the contour intersection method is less than 1.5%. For the imaginary part of the complex refractive index, the relative error of the iterative method is as high as 1.2%. However, the relative error of the contour intersection method is less than 0.5%. In general, the influence of the proportion of Au molar fraction on the inversion results of the iterative method is greater than that of the contour intersection method. The relative error in the inversion results of the iterative method is greater than the relative error in the inversion results of the contour intersection method. This indicates that the inversion accuracy of the contour intersection method is better than the traditional iterative method.

**Figure 7:**
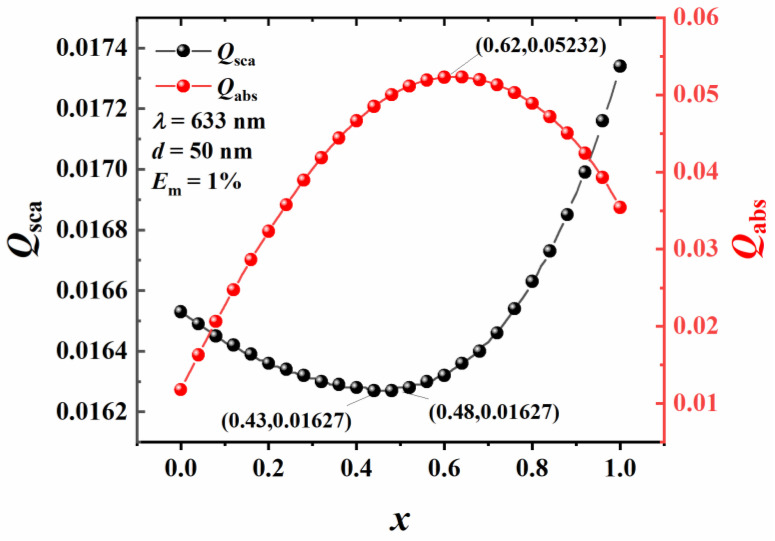
Scattering and absorption efficiencies of Au-Ag alloy nanosphere with respect to the Au molar fraction.

Particle size is an important parameter affecting particle light scattering and absorption characteristics. Therefore, the effect of particle size on the inversion of the complex refractive index of Au-Ag alloy nanospheres is analyzed. The step length is 0.001, the *E*_m_ is 1%, the incident light wavelength is 633 nm, *x* is 0.62, and particle sizes are 20 nm, 40 nm, 60 nm, 80 nm, and 100 nm. The selection of the Au molar fraction is based on the analysis of the variation of the scattering and absorption efficiencies of Au-Ag alloy nanoparticles, considering the Au component ratio and the experimental results of Besner, Meunier et al. [21], as shown in Figure 7.

**Figure 8:**
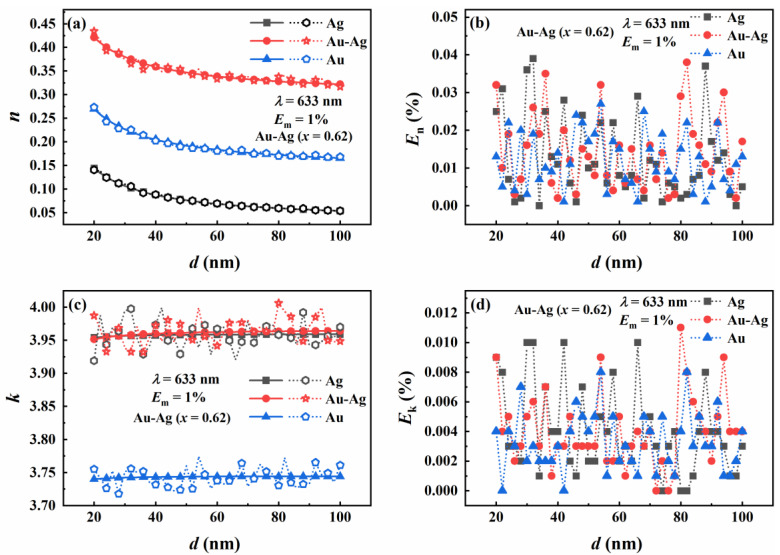
The effect of particle size on the real part (*n*) and imaginary part (*k*) of the refractive index inverted by the contour intersection method. (**a**) The inversion results of the real part of the refractive index where molar fraction of Au is 0, 0.62, and 1, respectively. (**b**) The relative errors in the inversion of the real part of the refractive index. (**c**) The inversion results for the imaginary part of the refractive index where molar fraction of Au is 0, 0.62, and 1, respectively. (**d**) The relative errors in the inversion of the imaginary part of the refractive index.

It can be seen from Figure 7 that the absorption efficiency of Au-Ag alloy nanoparticles increases first and then decreases with the increase of the Au molar fraction. Moreover, the absorption efficiency of Au-Ag alloy nanoparticles reaches the maximum at 0.62. The scattering efficiency decreases first and then increases with a minimum between 0.43 and 0.48. When the gold molar fraction in the alloy is higher than 0.4, the oxidization of most of the nanoparticles is quenched, which effectively inhibits the release of toxic silver ions into the solution. Alloy nanoparticles with 0.62 Au molar fraction in Au-Ag alloy are easy to manufacture and conducive to mass production. Therefore, in this work, 0.62 Au molar fraction is selected. The relevant complex refractive index is inverted by the contour intersection method, the results of which are shown in Figure 8.

In Figure 8, three precious metals, pure silver, gold–silver alloy, and pure gold, with Au molar fractions of 0, 0.62, and 1, are compared and analyzed. It can be seen from Figure 8a that the real part of the complex refractive index decreases with the increase in particle size. Moreover, the real part of the complex refractive index of gold–silver alloy is greater than that of pure gold. Interestingly, the real part of the complex refractive index of pure gold is greater than that of pure silver. Figure 8b shows the relative errors of the real part of the complex refractive index. It can be seen that the relative errors in the real part of the complex refractive index of pure gold are less than that of the alloy and pure silver as the particle size increases. The relative error of the real part of the complex refractive index of the alloy is greater than that of pure silver, and the relative errors of the two are <4%. The reason is that the *Q*_sca_ of alloy nanoparticles increases with particle size and is larger than that of pure gold and pure silver. The influence of particle size on the *Q*_sca_ of alloy nanoparticles is more significant, resulting in a large relative error. Figure 8c shows that the imaginary part of the complex refractive index changes minutely with increasing particle size.

Additionally, the real part of the complex refractive index of gold–silver alloy and pure silver is greater than that of pure gold. It shows that the particle size dramatically influences the real part of the complex refractive index but has little effect on the imaginary part of the complex refractive index. The proportion of silver in gold–silver alloy greatly influences the imaginary part of the complex refractive index. Figure 8d presents the relative errors in the inversion of the imaginary part of the complex refractive index. Figure 8d shows a similar trend to those in Figure 8b. The relative errors in both figures are less than 1.2%. The difference in the relative errors of the real and imaginary parts of the complex refractive index is that the particle size has different effects on the particle scattering and absorption efficiencies, resulting in different accuracy of the inversion results.

**Figure 9:**
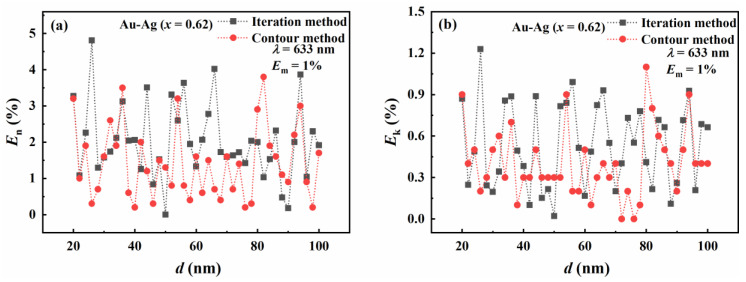
The relative error in the inversion results of the contour intersection and iterative methods with respect to particle sizes. The relative errors of the real part (**a**) and imaginary part (**b**) of the complex refractive index. The molar fraction of Au is 0.62.

Figure 9 compares the inversion accuracies of the contour intersection and traditional iteration methods to further explore the influence of particle size. For the real part of the refractive index, it can be seen from Figure 9a that the relative error of the contour intersection method is less than 4%. The relative error of iterative inversion is less than 5%. For the imaginary part of the refractive index, it can be seen from Figure 9b that the contour intersection method has the best inversion result, in the range from 25 nm to 80 nm. The relative error in the contour intersection method inversion is less than that in the iterative method inversion. In general, in the particle size range from 20 nm to 100 nm, the relative error in the iterative method inversion is greater than that of contour intersection inversion. This shows that in the range from 20 nm to 100 nm, the inversion accuracy of the contour intersection method is better than the traditional iterative method under the same conditions.

**Figure 10:**
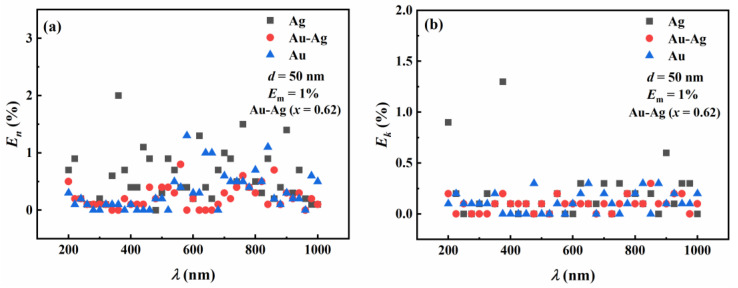
Relative errors in the refractive indices of Ag nanoparticles, Au-Ag alloy nanoparticles, and Au nanoparticles with respect to incident wavelengths after inversion by the contour intersection method, where x is 0.62 for Au-Ag alloy. (**a**) Relative errors in the inversion of the real part of the refractive index. (**b**) Relative errors in the inversion of the imaginary part of the refractive index.

Figure 10 shows the relative errors in the complex refractive index with respect to the incident wavelength for Ag, Au-Ag, and Au nanoparticles using the contour intersection method. The particle size for all three materials is 50 nm. The incident wavelengths range from 200 nm to 1000 nm, and the measurement error is 1%. The molar fraction of Au in Au-Ag alloy nanoparticles is 0.62, with *x* equal to 0.62. The results show that the relative errors in the inversion of the real parts of the complex refractive indices for all three nanoparticles are less than 2.2%. Similarly, the relative errors in the inversion of the imaginary parts of the complex refractive indices for all three nanoparticles are less than 1.5% with respect to the incident wavelengths. The influence of the incident wavelength on the inversion results is relatively small.

As shown in Table 3, Au-Ag alloy nanospheres with a value of *x* equal to 0.62 and diameters of 50 nm were irradiated by visible light at 633 nm wavelength. Table 3 gives the original values of the real and imaginary parts of the complex refractive index, the inverted complex refractive index values, their relative errors, and the time required for taking different steps in the complex refractive index range. From Table 3, it can be seen that the relative errors become progressively larger as the step-size increases. The relative error of the inversion results can be reduced by selecting smaller step sizes in the inversion process. However, accuracy can then be acquired at the expense of time.

The actual measurement process inevitably introduces errors, such as measurement errors and calculation errors. The particle diameter of 50 nm, step length of 0.001, Au molar fraction of 0.62, and wavelength range from 200 nm to 1000 nm were selected for inversion. The complex refractive indices of Au-Ag alloy nanoparticles with *E*_m_ of 0%, 1%, 5%, and 7% were inverted by the iterative and contour intersection methods, and the relative errors were analyzed.

**Figure 11:**
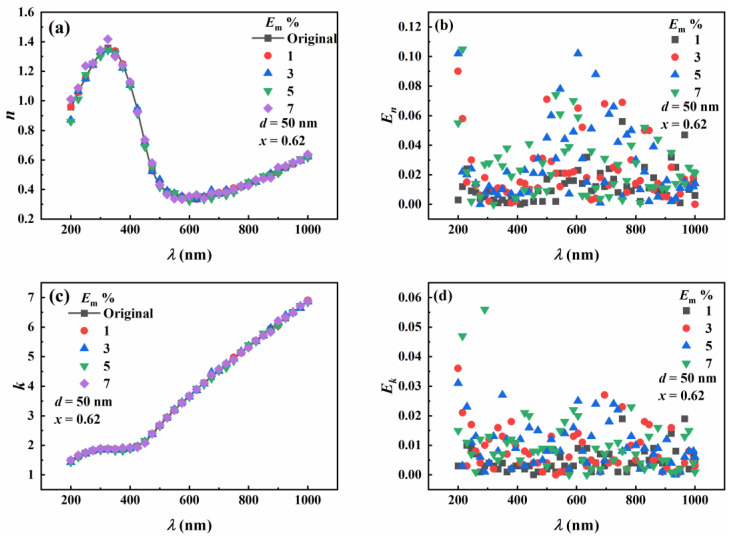
The effect of measurement error on the inversion of refractive index by contour intersection method. (**a**) Inversion of the real part of the refractive index with a molar fraction of Au 0.62. (**b**) The relative error in the inversion of the real part of the refractive index. (**c**) The inversion of the imaginary part of the refractive index. (**d**) The relative error in the inversion of the imaginary part of the refractive index.

Contour-intersection-method inversion results are shown in Figure 11, where (a) and (c) present the inversion results of real and imaginary parts of the complex refractive index. It can be seen from Figure 11a,c that the real part of the complex refractive index increases first, then decreases, and then increases with the increase of wavelength. On the other hand, the imaginary part increases with the increase of wavelength. The inversion results become inaccurate with the increase of measurement errors. Figure 11b shows the relative errors in the inversion of the real part of the complex refractive index. It can be seen that the relative errors in the inversion are larger when the incident light wavelength is close to 200 nm and 600 nm. When *E*_m_ = 5%, the relative error in the real part of the complex refractive index is about 10%. The relative error fluctuates when *E*_m_ > 5%, and the inversion results become unstable. The possible reason is the smaller wavelength, which dramatically influences the particles’ scattering and absorption characteristics. Figure 11d manifests the relative error in the imaginary part of the complex refractive index, which has a trend similar to that of Figure 11b. The relative error in the imaginary part of the complex refractive index is below 3% when *E*_m_ < 5%.

**Figure 12:**
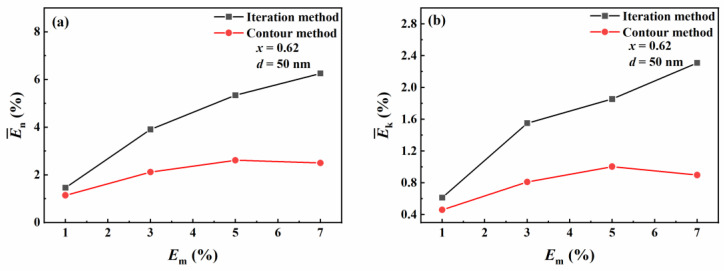
The average relative errors in the inversion results of the contour intersection method and the iterative method with respect to *E*_m_. The average relative errors in the real (**a**) and imaginary (**b**) parts of the refractive index at an Au molar fraction of 0.62.

Figure 12 compares the inversion results of the contour intersection method and the traditional iterative method with respect to *E*_m_ (measurement errors). It can be seen from Figure 12a that for the real part of the complex refractive index, the mean relative error of the inversion results from the iterative method increases with the increase in the measurement error. The mean relative error of the inversion results from the contour intersection method also has the same trend when *E*_m_ < 5%. However, when *E*_m_ > 5%, the mean relative error of the real part of the complex refractive index decreases. It can be seen from Figure 12b that for the imaginary part of the complex refractive index, the trend is similar to the real part of the refractive index. The overall analysis shows that the average relative error of the inversion results from the contour intersection method is less than that of the inversion results from the iterative method. When *E*_m_ > 5%, with the sharp increase in the number of intersection points, the inversion result values show unpredictable volatility. An iterative method is a method of checking for the solutions against output conditions in a specific range. The increase in the measurement error largely depends on the selection of the starting parameters. Therefore, the influence of measurement error in the iterative method is stronger than that of the contour intersection method.

This work demonstrates the feasibility and accuracy of contour intersection method for determining the complex refractive index of a material using computation experiments rather than actual measurements. The proposed method involves a combination of two techniques: the inversion method, which uses numerical algorithms to invert the complex refractive index from measured scattering and absorption data, and the contour-crossing method, which analyzes the analytic continuation of the complex refractive index in the complex plane to retrieve the original refractive index.

## 4. Conclusions

In this work, the feasibility of inverting the complex refractive index of Au-Ag alloy nanospheres by light absorption and scattering is quantitatively studied using the Mie theory and size-dependent dielectric function. According to the relationship between light scattering, absorption, and complex refractive index, the complex refractive index of Au-Ag alloy nanospheres is inverted by the contour intersection method. The constraint method of determining the unique solution from multiple effective solutions was introduced in the contour intersection method. The effects of gold molar fraction, particle size, measurement error, and step size on the inversion results were quantitatively analyzed during the inversion process. The feasibility and accuracy of the inversion of the complex refractive index of Ag nanoparticles, Au-Ag alloy nanoparticles, and Au nanoparticles by the contour intersection method were analyzed in the incident wavelength range of 200 nm–1000 nm. Finally, the complex refractive indices of gold–silver alloy nanoparticles inverted by the contour-crossing method were compared with the original values. Moreover, the inversion accuracy was also compared to the conventional iterative method. The speed of inversion can be improved, and the accuracy of inversion results can be ensured if step sizes are in the range of 0.001–0.01 and measurement error is less than 5%. Under the same conditions, the inversion accuracy of the contour intersection method is better than that of the traditional iterative method. The complex refractive index of Au-Ag alloy nanoparticles can be obtained using the contour intersection method. In this paper, preliminary results have been achieved by inverting the refractive index of Au-Ag alloy nanoparticles. Our further research will focus on developing visualization software for the inversion of the complex refractive index of particles and improving the inversion algorithm to obtain high-accuracy inversion results easily and quickly.

## Figures and Tables

**Table 1:** Fitted parameters of the dielectric function of Au-Ag alloy.

Metal	Au	AuAg_5050_	Ag
*ω*_p_/eV	8.9234	9.0218	8.5546
*Γ*_p_/eV	0.042389	0.16713	0.022427
*ε*_∞_	2.2715	2.2838	1.7381
*ω*_g1_/eV	2.6652	3.0209	4.0575
*ω*_01_/eV	2.3957	2.7976	3.9260
*Γ*_1_/eV	0.1788	0.18833	0.017723
*A*_1_	73.251	22.996	51.217
*ω*_02_/eV	3.5362	3.3400	4.1655
*Γ*_2_/eV	0.35467	0.68309	0.18819
*A*_2_	40.007	57.540	30.770

**Table 2:** The inversion results from the contour intersection and the iterative methods with a wavelength of 633 nm and nanoparticle diameters of 50 nm. The value of x is 0.62.

Method	*n*_o_	*k*_o_	*n*_i_	*k*_i_	*E_n_* (%)	*E_k_* (%)
Contour intersection method	0.34677	3.96138	0.34639	3.96082	0.11	0.1
Iterative method	0.34677	3.96138	0.33956	3.94333	2.08	0.46

**Table 3:** The inversion results and times of the contour intersection method with different complex refractive index steps for an incident wavelength of 633 nm, diameters of 50 nm, and *x* equal to 0.62.

Step Size	*n*_o_	*k*_o_	*n*_i_	*k*_i_	*E_n_* (%)	*E_k_* (%)	Time (s)
0.0005	0.34677	3.96138	0.34684	3.96167	0.021	0.007	632.37
0.001	0.34677	3.96138	0.34831	3.96601	0.444	0.117	50.81
0.005	0.34677	3.96138	0.34927	3.96617	0.720	0.121	1.40
0.01	0.34677	3.96138	0.35052	3.96992	1.079	0.216	0.81
0.05	0.34677	3.96138	0.33770	3.93061	2.616	0.777	0.73
0.1	0.34677	3.96138	0.35882	3.99743	3.473	0.910	0.66

## Data Availability

The data presented in this study are available in article.
